# Rural Gambian women's reliance on health workers to deliver sulphadoxine – pyrimethamine as recommended intermittent preventive treatment for malaria in pregnancy

**DOI:** 10.1186/1475-2875-8-25

**Published:** 2009-02-12

**Authors:** Loretta Brabin, Elizabeth Stokes, Isatou Dumbaya, Stephen Owens

**Affiliations:** 1 Academic Unit of Obstetrics & Gynaecology, University of Manchester, Manchester M13 OJH, UK; 2 Medical Research Council, Keneba Field Station, Keneba, the Gambia

## Abstract

**Background:**

The use of most anti-malarial medications is restricted during pregnancy, but two doses of sulphadoxine-pyrimethamine are recommended after the first trimester as intermittent preventive treatment in pregnancy (IPTp). In The Gambia, only 32% of women receive two doses and very little research has been conducted on women's awareness of drug safety during pregnancy. The objective of this paper was to assess whether rural Gambian women were aware of the importance of the timing of the two-dose IPT dose schedule and its relevance to drug safety.

**Methods:**

This was a qualitative study in which 41 interviews and 16 focus group discussions with women, adolescents, men and traditional birth attendants were conducted. A generic qualitative approach was used to generate a theory as to why women might not participate in IPTp as recommended.

**Results:**

Although most women used calendar months to count their stage of pregnancy, these months did not correlate with their concept of foetal development. Foetal growth was described following Islamic tradition as water, clot, piece of meat and human being, although there was little consensus about the order or timing in which these stages occurred. Common signs and conditions of malaria were known. Women were anxious about miscarriage and recognized that some medicines should not be taken in the first trimester, but were urged by men and traditional birth attendants to attend for antenatal care in the first trimester to "start treatment." General knowledge about the purpose of pregnancy medications and when they should be taken was poor among both men and women. One important result was that women relied entirely on health workers to provide safe drugs, at the correct time.

**Conclusion:**

Women did not have relevant information to judge the safety and appropriate timing of pregnancy drugs, which made them over-reliant on health workers. They should be encouraged to date their own pregnancies in culturally relevant terms and to anticipate when and which medications they should receive.

## Background

Many anti-malarial drugs have not been licensed for use in pregnancy because their risk has not been assessed in controlled trials and some are potentially embryotoxic [1]. In most African countries, including The Gambia, sulphadoxine-pyrimethamine (SP) is recommended during the second and third trimesters of pregnancy for intermittent preventive treatment (IPT) in areas of moderate to high malaria transmission [2]. SP has a favourable safety profile during pregnancy despite some concerns about severe cutaneous reactions and teratogenesis, although it is not recommended for HIV-positive women on cotrimoxazole prophylaxis for whom there is a higher risk of severe adverse reactions [3]. Use of anti-folate agents like SP is also contra-indicated in the first trimester because of the risk of neural tube defects. A postulated association between SP and kernicterus has not been observed in practice. The risks of malaria to mother and foetus are judged to outweigh potential risks of SP toxicity, and policies and protocols governing its use are intended to protect pregnant women and their babies from IPTp exposure in the first trimester [2].

Despite this, without good training and close supervision of health care workers, there is a risk that women will be treated incorrectly. In the absence of pregnancy registers, the size of this risk is still largely unknown [4]. Nor has there been much research on women's awareness of drug safety issues in early pregnancy. Inadvertent exposure to drugs that are contra-indicated in early pregnancy could arise if women were unaware of, or failed to disclose their pregnancies. This hypothesis was investigated qualitatively among Gambian rural women in an earlier publication, in which it was reported that women consciously sought to hide their pregnancies as a way of retaining some control in their lives and avoiding malign gossip [5]. This resulted in delayed attendance for antenatal care (ANC). For the same reason, women with clinical malaria sometimes chose not to disclose an early pregnancy when seeking out-patient treatment. As health workers in out-patient clinics frequently failed to inquire about pregnancy, this potentially exposed women to the risk of being given medications that might have been contra-indicated [5].

In general, ANC is the main route for obtaining IPTp and late ANC attendance is one of the most common reasons given to account for low IPT coverage [6]. Another is that women attend ANC during the first trimester, when they cannot be treated and do not return for their first IPTp dose at the recommended time (between 14 and 34 weeks) [7]. Failure to return suggests that women may not appreciate the importance of the timely administration of IPTp and are not pro-active in requesting it. In the Gambia, 98% of women attend ANC at least once during pregnancy [8], a half visit at least four times and about 25% first present before 20 weeks [9]. Despite this high level of contact with health facilities and the provision of free IPTp, coverage for two IPTp doses among women aged 15–49 was reported to be just 32%, with little difference between urban and rural areas or in relation to education or wealth indices [8]. In view of this low coverage, data from the earlier study on pregnancy disclosure has been analysed to assess women's awareness of the need for timely IPTp and is reported in this paper.

## Methods

Ethical approval was obtained from the University of Manchester's Committee on the Ethics of Research on Human Beings and the Gambian Government/Medical Research Council (MRC) Joint Ethics Committee.

### Participants and study area

As previously described [5] the study was conducted between February and November 2007 in rural Kiang West, The Gambia, and participants were mainly Muslim and belonged to the Mandinka tribal group. The research took place in three villages (Jiffarong, Dumbuto and Janneh Kunda) that were eight, 30 and 40 kilometres away from the UK Medical Research Council (MRC) field station at Keneba. Training interviews were undertaken in nearby Manduar. ANC in each village was provided by MRC/government trekking teams, assisted by MRC Keneba midwifery and medical staff. Coverage of ANC by skilled personnel (doctor, nurse, or midwife) is relatively high in The Gambia and about half of the female population delivers in a health facility [8]. Standard of Practice Guidelines used in West Kiang at the time of the study instructed nurses/midwives to offer at least two doses of SP during pregnancy and advised that SP was considered safe between 14–34 weeks but should not be given in the first trimester or after 34 weeks. The recommended dose was three tablets, with the 1^st ^dose at 18–28 weeks and the 2^nd ^dose at 30–34 weeks, with doses spaced four weeks apart, to be taken under direct observation. The instructions were also to screen pregnant women at prenatal visits for fever or other symptoms of malaria, to make a thick film if symptomatic, then to treat with SP and chloroquine. Otherwise nurses/midwives were to encourage women to use insecticide-treated bed nets and take other measures to avoid mosquito bites. To prevent anaemia all women were given daily supplementation tablets of combined iron and folate. Other drugs routinely used were paracetamol, methyldopa, diazepam, amoxicillin/ampicillin and metronidazole.

Using a list generated from the MRC database, nulliparous and parous women were consecutively approached for either in-depth interviews or focus group discussions (FGDs). Men who were regarded as senior in terms of village authority or who practiced polygyny were recruited by community health workers, as were traditional birth attendants (TBAs). Women who were participating in a MRC micronutrient trial were excluded to avoid any concerns about their supplementation. The consent of the *alkalo *(village head) was sought to approach individuals living in the village. Individual participants gave written (signature or thumb print) consent. Four FGDs were held with younger married women, four with older married women, three with adolescent girls, one with traditional birth attendants and four with men (16 in total). A total of 41 in-depth interviews were conducted.

### Interviews

Audio-taped interviews and FGDs with women were conducted by a Gambian nursing-assistant (ID), and those with men by a male field worker. The sessions took place in a health centre or a village compound in which privacy could be maintained. Observations about malaria in pregnancy and the use of anti-malarials were recorded within a topic guide that covered broader pregnancy issues, but included women's use of medication during pregnancy. All interviews were attended by ES, and ID was trained not to ask questions that would make women anxious about drugs (eg foetal abnormalities). During training, questions were asked in English and translated into Mandinka to monitor the interview content, but once the quality of interviewing was satisfactory, interviews were conducted solely in Mandinka. ES and ID reviewed each session, listening to the tapes, which ID translated. This allowed ES to provide feedback and to identify emerging topics to be explored in later interviews. The transcripts were sent to a Gambian commercial company for formal translation. Those judged to be of unsatisfactory quality were returned for re-translation.

### Data analysis

Transcripts were read independently by ES and LB. A generic qualitative approach was used [10]. The theoretical position was derived from medical ethics, which informed the research topics (e.g. reasons why women remained unaware of the significance of the IPTp schedule), and led to a theory as to why women might not receive IPTp as recommended. Actions that influenced IPTp treatment were systematically coded in an iterative process that allowed categorisation of sub-themes. Interviews, FGDs and subgroup data were compared and repeated and discordant themes identified. The process continued until both researchers were satisfied with the explanatory process. In this report, combined data from FGDs and interviews are presented and main themes illustrated by verbatim quotations translated from Mandinka to English.

## Results

### Measuring stage of pregnancy

When asked how they measured their pregnancy, most women said "*by moon" *and/or calendar months. They and their husbands monitored their menstrual cycle because they were not allowed to sleep with their husbands, pray or do other household tasks when menstruating. A men's focus group identified one of the problems of identifying stage of pregnancy when relying on calendar months: *"There are some women who will see their period at the beginning of the month, others will see it around day fifteen, others will have it around day twenty five .... In a case where pregnancy reduces *(ie the period occurs early in the month), *some women may go up to 10 or 11 months before they deliver." *[JKMFG-4].

### Stages of foetal growth

Foetal growth was described following Islamic tradition as water, clot, piece of meat and human being. These stages were recognized by all men and women of different ages. Men were more consistent about the order of these events but not their timing. Among women there was little consensus about the timing or order in which these stages occurred (Table 1, a-d), and as an understanding of basic embryology was not part of TBA training, their views were similar to those of other older women. Adolescent girls were particularly vague about foetal growth stages. "Quickening" or the first movement of the foetus, which health workers partly relied on as an indicator for giving the first IPTp dose, was not consistently associated with any particular stage of pregnancy by women. The baby was described as kicking or *"shaking*" at various times after three months (Table 1, a-d). Generally the foetus was not regarded as human before the fourth month.

**Table 1:** Description of stages of foetal growth by different groups

**MONTH**	**YOUNG WOMEN 1a**	**OLDER WOMEN 1b**
	"Oh, I don't have any idea about that." (JKW1-1)	

**1**	"It looks like a piece of meat." (DYWI-6)	"It is in the semen stage" (JKOWFG-1) "It will remain water for one month before it will turn into blood." (MOWFG-1) "It is just like water." (JKOWI-6)

**2**	"It has a grey colour."(DYWI-6)	"It is in a liquid stage" (JKOWFG-1) "It appears like a clot." (JKOWI-6)

**3**	"It looks like a lizard."(DYWI-6) "It looks like pus....The baby is kicking on one side...It moves but the movement is not powerful." (DYW1-2) "It is in a blood-like state." (DYWI-4)	"It is in a clot form." (JKOWFG-1) (JKOWI-2) "If the foetus is a female, it looks like a tortoise. If it is a male, it looks like a lizard." (JKOWFG-1)

**4**	"It is nearly mature"(DYWI-6) "Limbs form" (JKYWI-4) "From 4–6 months it kicks from all directions" (DYW1-2)	"It starts kicking at four months" (JKOWFG-1) "At four months it will no longer be blood or a piece of meat. It will be a human being. You will know whether the baby will be a boy or a girl." (MOWFG-1) "At four months it is just like a bulk of blood." (JKOWI-2) "Limbs will start to appear." (JKOWI-6)

**5**	"I think it stays like that until 5–7 months. Then God breaks it into two pieces and reconstructs it again"(DYWI-6) "From five to six months, all features of a human being are developed.....but nine months is the time that the baby is fully matured." (JKYWI-4)	"When the baby has been five months in the stomach, the stomach will shake." (MOWFG-1)

**6**	"It looks like a piece of meat."(JKYWI-4)	"Within six months the baby is close to a human being." (JKOWFG-1) (JKOWI-2) "At six months the stomach will shake again" (MOWFG-1)

**8**	"It is fully matured in all aspects"(DYWI-6)	"At eight or nine months the stomach will shake and you will know that God's time has come for you to deliver." (MOWFG-1)

**9**	"It is now a complete human being. At this stage, if you feel hungry, you see the baby start moving"(DYWI-6)	

**MONTH**	**ADOLESCENTS 1c**	**MEN 1d**

		"Pregnancy is never steady.. it has stages, from semen to blood clot, until it departs from there, that will equal to 120 days." (JKMFG-4)

**1**	"It looks like a piece of meat" (DAFG-1) "It appears like a blood clot" (JAFG-3)	"The pregnancy is just semen" (JJMFG-1) "At 7 days, pregnancy is a clot" (JKMFG-3)

**2**	"Clot" (DAFG-1)	"Blood clot" (JJMFG-1) "It begins to have human shape." (JKMFG-3)

**3**	"I don't know." (DAFG-1) "It then looks like a small frog" (JAFG-3)	"It is not at a human stage, it is like blood." (JKMFG-3)
	
**4**	"By then it is an egg." (DAFG-1)	"The blood clot becomes firm." (JJMFG-1) "I cannot tell you anything about that". I heard women saying that there is movement at four months, but I don't know.(JKMFG-2) "At four months, things come together." (JKMFG-3)

**5**	"It is about to become a human being." (DAFG-1)	"A piece of meat" (JJMFG-1) "Limbs are forming" (JKMFG-3) "At this stage it is now human." (JKMFG-3)

**6**	"It starts moving" (DAFG-1)	"Limbs are forming" (JKMFG-3) "It is now a complete human being" (JKMFG-3)

**7** **8** **9**		"At seven months it is mature" (JKMFG-3) "It starts having limbs" (JJMFG-1) "At 7 months there is a soul in the body, but at 8 months the soul is suspended from the body. By 9 months it reunites with the body again" (JJMFG-1) "It turns into a human being" (JAFG-3)

### Effects of malaria on the foetus

Men and women were able to list the common signs of malaria such as shivering, fever, vomiting, joint pain, general body weakness. They knew it caused anaemia and pre-term birth and that bed nets offered protection. They had some idea of the mechanisms by which malaria caused illness in a pregnant woman and foetus. One man, recounting how his brother-in-law's pregnant wife had to go to hospital with malaria, had been told that malaria reduced the blood by 60%, but was puzzled and asked *"How can that be possible?" My brother in-law said different parts of the body start pulling against each other, and that affects the blood." *[JKMFG-3]

Malaria was considered to affect the foetus indirectly. When young women were asked, *"Do foetuses get sick in the womb?"*, one replied *"Yes, it gets sick. It may be that it does not have the parasites but if the mother is not eating good food, that can make it get ill." *[MYWFG-1]

### Perceived safety of medications during pregnancy

Women were able to understand the concept that some drugs were contra-indicated early in pregnancy. There was general agreement that a local herb (jambakasala) "*cleaned the baby" *in utero and was safe even in the first month of pregnancy unlike another herbal remedy (katijangkumou) which could not be taken till later. Women named chloroquine/nivaquine as a western drug to be avoided during the first trimester because it was bitter, a characteristic associated with abortifacients. Some of the men thought that paracetamol also caused abortion.

TBAs actively encouraged women to go to ANC in the first trimester, *"because doctors like that, so that she (the mother) can start treatment." *[DTBAI-1] Men uniformly advised along similar lines, noting *"You should start taking your medication at one to two or three months. That way they (health workers) can help you*." [JKMFG-4] Despite this injunction, men had very little knowledge of the drugs given to women, other than describing their colour, and some idea that red ones were for blood. One said,

*"My advice is to go to the clinic to see a nurse. Those people will tell her what to do and they will give her some medication." *[JKMFG-3].

A similar view was as follows:

"*Well, as human being we take those drugs with the hope that they are useful. I don't think if they are useless, the woman will have any cause to take them" *[JJMFG].

Women were equally unclear about the drugs they were given and accepted their safety if given by a nurse or doctor at ANC. One group of TBAs thought that women were given "*an injection for Jarrara *"(cerebral malaria) [JOLITBA-1], which presumably was the routine tetanus toxoid vaccination. When asked *"What are the medicines for?" *typical replies were:*"I don't know, but they said if I take them, my dizziness and shaking will go away" *[JKYWI-3] and "*The red tablet is for blood increase. The white tablet is for good health and the yellow tablet also serves the same purpose – strength and good health." *[JKYWI-4]

### Knowledge of IPTp dosing schedules

The fact that malaria causes anaemia and that iron prevents anaemia led to some confusion about the difference between anti-malarial and iron treatments. When asked about the month when IPTp was first given, one comment was as follows:

*"On one occasion I was at four months, some other occasions, if I bought a ticket *(for ANC registration) *and I had normal health, they wouldn't give it to me. But as time went on, they would check my blood to see whether it was going up or down. Then they would start giving it to me." *[JK0WFG-1 Women's reports of how many doses of IPTp they had received were unreliable. Some older women claimed they had received as many as five doses. [JKOWFG-1] Younger women generally knew that they should get two doses, but even this was disputed, as shown in the following conversation:

"How many times do you take your malaria medication?"

"Three times"

"Me, twice"

*"It was supposed to be three. If you take anti-malarial drugs during the first three months, the effects of the drugs lessen the severity even if you have parasites in your blood. The second dose provides you longer protection. If you complete the third dose, even if you have parasites in the blood, they will not harm you." *[MdYWFG-1]

Women consistently said they did not know when to take IPTp.

"How did you know it is time for you to take the second dose?"

"I don't know. I went to the Health Centre. I was given it by them when the time was right."

"Did you know, or have any idea when you should take the anti-malarial drug?

*"No idea." *[JWYI-1]

When asked how she knew it was right time for IPTp, another said *"When I came here for the first time they checked me and I was given the first dose. Then I came for the second, it was the same. I was given a second dose, but I cannot tell if it was the right time." *[JWYI-2]

## Discussion

These results highlight a general ignorance of the importance or relevance of the IPTp schedule within this rural Gambian population, which is likely to reflect, at least in part, a paradigm of foetal development that was not based on weeks or trimesters. It also reflected a very confused understanding of the different drugs given to pregnant women and lack of awareness that some (such as haematinics), but not all medications, may be used safely in the first trimester. The main consequence was that women were entirely dependent on the ANC nurse to give IPTp correctly, as women and their husbands firmly believed that unsafe drugs would not be dispensed by health workers. In other respects, this Gambian community was quite well informed about the causes and effects of malaria, which is perhaps not surprising given that the MRC has conducted malaria studies in this country over many years and malaria prevalence has declined following the community uptake of the various control strategies that have been introduced [11]. This may also partly account for general acceptance by the community that IPTp was safe, although elsewhere, side effects have caused anxiety and fears [12,13].

The results do not provide a comprehensive account of the determinant of IPTp effectiveness as this study was not specifically designed to investigate IPTp uptake. Questions on IPTp were asked because it was one of the most common drugs to which women in this setting were exposed, but use of anti-malarial drugs was only part of a more detailed interview schedule encompassing pregnancy recognition and disclosure, and its potential consequences for inadvertent drug exposure. Qualitative research is also not generalisable but these results are relevant for those working in other Islamic communities that share similar concepts of foetal development [14]. Even then, local variations based on autochthonous traditions may be evident [15]. In Islam, the foetus is described as progressing through a series of changes leading to "ensoulment", when an angel breathes spirit into the foetus at 120 days and it becomes a human being, with legal rights [16,17]. Since women did not conceptualize their pregnancies in weeks and trimesters, they may have had difficulties in understanding the timing of IPTp. Sometimes women are asked about "quickening", understood to mean the first maternally-detected movements of the foetus, which typically occur around 18 weeks gestation. However, women who conceptualize the foetus as a "clot" may not relate to this indicator as expected. Some Nigerian research provided an example of how an educated secondary school educated Yoruba mother had learnt to correlate accurately her account of foetal development based on Islamic concepts with calendar months [14]. This demonstrates that it should be feasible to teach women to date their own pregnancies in culturally relevant terms. As well as helping mothers to better understand the physiology of pregnancy, it could be used to inform women of the purpose and timing of IPTp, and to explain why some drugs such as IPTp should be avoided, especially in the first trimester. As in other cultures [18], women partly understood this concept and avoided some local and western medications during early pregnancy, but this was not linked to any factual knowledge about foetal growth and how some drugs can harm the developing foetus.

Women's reliance on health workers to administer drugs correctly was understandable as women were accustomed to obeying authority figures, but this practice carries risks that could be reduced if they were better informed. Although the situation has not been assessed in The Gambia, erratic timing of IPTp doses has been identified as an important issue in other countries [19]. In one Kenyan study, only 15.7% of the women who had two IPTp doses had received them at the correct time [6]. In Malawi, it was reported that health workers were not adhering to exclusion of first trimester pregnancies [18]. All women were administered IPTp during their first clinic visit, irrespective of the week of pregnancy, which ranged from 10 to 36 weeks, and nearly a quarter of women said they were told to take it in the first trimester. In Uganda 9% of women also received IPTp in the first trimester [20]. The Gambian women in the present study seemed unaware that IPTp was contraindicated in the first trimester. Unlike chloroquine and some other bitter medicines, it was not spontaneously mentioned as a drug to avoid because of the risk of miscarriage. A common factor shared by women in most cultures is fear of an early pregnancy loss, which was one of the reasons that Gambian women delayed ANC attendance [5]. In Kenya, mothers who had experienced a child death were significantly less likely to have received SP and its use was associated with miscarriage [13]. There is scope for a great deal of misunderstanding when women do not comprehend why some drugs can be used safely in later pregnancy, but not in the early stages. There are similar issues around the number of recommended doses. These Gambian women, like some Kenyans [13] did not know how many IPTp doses they should receive. Some women claimed to have been given multiple doses, but this may have been because they confused malaria and haematinics. In settings where monthly IPTp has been recommended, this could lead to excessive dosing. A median of five doses was administered in one study of IPTp in HIV-positive women [21]. No adverse events in mothers or newborns were observed, but as there was no difference in pregnancy outcomes between HIV-negative women who receive multiple, as opposed to two doses of IPTp, higher frequency dosing had little to recommend it. There are also reports of women receiving IPTp at an interval of less than four weeks [6]. These risks remain as long as women, and those who advise them (eg. their husbands) are so confused about pregnancy medication.

## Conclusion

This study has highlighted how important it is for women to understand the stages of pregnancy, as well as which drugs cannot be safely taken in early pregnancy and why. Studies should be conducted to confirm that women have made sense of this information and are confident in their use of IPTp. This would go some way to helping them avoid untimely administration of IPT and other medications. This process should run alongside health worker training, which is essential, but only part of the solution [22].

## Abbreviations

FG: Focus group; I: Interview; YW: Younger woman; OW: Older woman; A: Adolescent; M: Male; TBA: Traditional birth attendant; J: Jiffarong Village; D: Dumbuto Village; JK: Janneh Kunda Village; Md: Manduar.

## Competing interests

The authors declare that they have no competing interests.

## Authors' contributions

LB designed the study, analysed some of the data and wrote the paper. ES conducted the research in the field and analysed the data. ID helped develop the research methodology and conducted the interviews. SO facilitated and advised on the study, reviewed drafts of the manuscript and co-chaired the health workers' workshop.
